# No changes in functional connectivity during motor recovery beyond 5 weeks after stroke; A longitudinal resting-state fMRI study

**DOI:** 10.1371/journal.pone.0178017

**Published:** 2017-06-08

**Authors:** Tanja C. W. Nijboer, Floor E. Buma, Caroline Winters, Mariska J. Vansteensel, Gert Kwakkel, Nick F. Ramsey, Mathijs Raemaekers

**Affiliations:** 1 Utrecht University, Department of Experimental Psychology, Helmholtz Institute, Utrecht, The Netherlands; 2 Center of Excellence for Rehabilitation Medicine, University Medical Center Utrecht and De Hoogstraat Rehabilitation, Utrecht, The Netherlands; 3 Brain Center Rudolf Magnus, University Medical Center Utrecht, Utrecht, The Netherlands; 4 Amsterdam Rehabilitation Research Center, Reade, Amsterdam, The Netherlands; 5 Neuroscience Campus Amsterdam, Vrije Universiteit Amsterdam, Amsterdam, The Netherlands; 6 Department of Rehabilitation Medicine, VU University Medical Center, Amsterdam, MOVE Research Institute Amsterdam, Amsterdam, The Netherlands; 7 Department of Physical Therapy and Human Movement Sciences, Northwestern University, Chicago, Illinois, United States of America; University of Glasgow, UNITED KINGDOM

## Abstract

Spontaneous motor recovery after stroke appears to be associated with structural and functional changes in the motor network. The aim of the current study was to explore time-dependent changes in resting-state (rs) functional connectivity in motor-impaired stroke patients, using rs-functional MRI at 5 weeks and 26 weeks post-stroke onset. For this aim, 13 stroke patients from the EXPLICIT-stroke Trial and age and gender-matched healthy control subjects were included. Patients’ synergistic motor control of the paretic upper-limb was assessed with the upper extremity section of the Fugl-Meyer Assessment (FMA-UE) within 2 weeks, and at 5 and 26 weeks post-stroke onset. Results showed that the ipsilesional rs-functional connectivity between motor areas was lower compared to the contralesional rs-functional connectivity, but this difference did not change significantly over time. No relations were observed between changes in rs-functional connectivity and upper-limb motor recovery, despite changes in upper-limb function as measured with the FMA-UE. Last, overall rs-functional connectivity was comparable for patients and healthy control subjects. To conclude, the current findings did not provide evidence that in moderately impaired stroke patients the lower rs-functional connectivity of the ipsilesional hemisphere changed over time.

## Introduction

Motor impairment is one of the most frequently occurring consequences of stroke [1]. Even though post-stroke recovery varies across patients and over time, prospective cohort studies have indicated that functional motor recovery at 6 months post-stroke onset is highly predictable within the first few days [2, 3]. The underlying mechanisms responsible for recovery, however, are not well understood. One mechanism that has been proposed to underlie functional recovery of upper limb paralysis is neuroplasticity. Spontaneous motor recovery appears to be associated with structural and functional changes in the motor network. Initial suppression of activation within the ipsilesional motor networks is gradually replaced by unilateral over-activation of the motor areas (as well as adjacent areas), during the initial stages of recovery [4, 5]. Optimal motor recovery coincides with patterns of activation comparable to those seen in healthy subjects [6], as well as with an overall normalised activity in secondary ipsilesional and contralesional sensorimotor areas post-stroke [7]for a review see [8]. These longitudinal changes in patterns of fMRI activity have also been reported during recovery of other modalities such as language [9], attention [10] and somatosensory impairments [11, 12].

The aim of the current study was to explore the time-dependent changes in resting-state (rs) functional connectivity in motor-impaired stroke patients, using rs-functional MRI. Rs-fMRI reflects the temporal synchrony of fMRI signals between remote regions, without the confounds associated with task-compliance or performance that can occur when using task-based fMRI. Previous studies indicated that rs-functional connectivity within either the ipsilesional primary sensorimotor cortex [13–15] or contralesional primary sensorimotor cortex [16] was decreased early after stroke, followed by a gradual increase during recovery up to near normal levels in those patients who also showed improvement in motor impairment. Here, we investigated in stroke patients: (1) overall time-dependent changes in rs-functional connectivity of motor networks; (2) the relation between magnitude of time-dependent changes in rs-functional connectivity and time-dependent changes in motor impairment as measured with the upper extremity section of the Fugl-Meyer Assessment (FMA-UE; [17]; (3) potential differences in rs-functional connectivity (at 5 weeks post-stroke onset) with healthy control subjects; and (4) potential changes in connectivity outside the motor system.

## Methods

### Participants

Thirteen stroke patients (eleven males) were included from the EXPLICIT-stroke Trial [18, 19], from August 2008 up to February 2013. Patients were included for this trial when they (1) had a first-ever ischemic stroke within the previous 2 weeks, verified by CT or MRI scan; (2) suffered from hemi- or monoparesis of the arm at baseline, determined by a National Institute of Health Stroke Score (NIHSS) item 5 of 4 point or less; (3) were able to make flexion-extension movements with the fingers or reach-to-grasp movements with the paretic upper-limb at 5 weeks post-stroke onset; (4) were aged between 18 and 80 years old; (5) were able to understand instructions as indicated by a Mini Mental State Examination score of 23 or higher (MMSE; [20]); (6) were able to sit for 30 secs without support; (7) demonstrated sufficient motivation to participate in an intensive rehabilitation treatment programme for at least 3 weeks; and (8) gave written informed consent to participate in the study. *Exclusion* criteria for this trial were (1) orthopaedic impairments of the upper extremities; (2) communication restrictions as indicated by a score of < 3 on the Utrecht Communication Observation (UCO; [21]); (3) botulinum toxin injections or other medication influencing the function of the upper-limb; and/or pacemakers or other metallic implants incompatible with the 3T MRI scanner.

**Fig 1:**
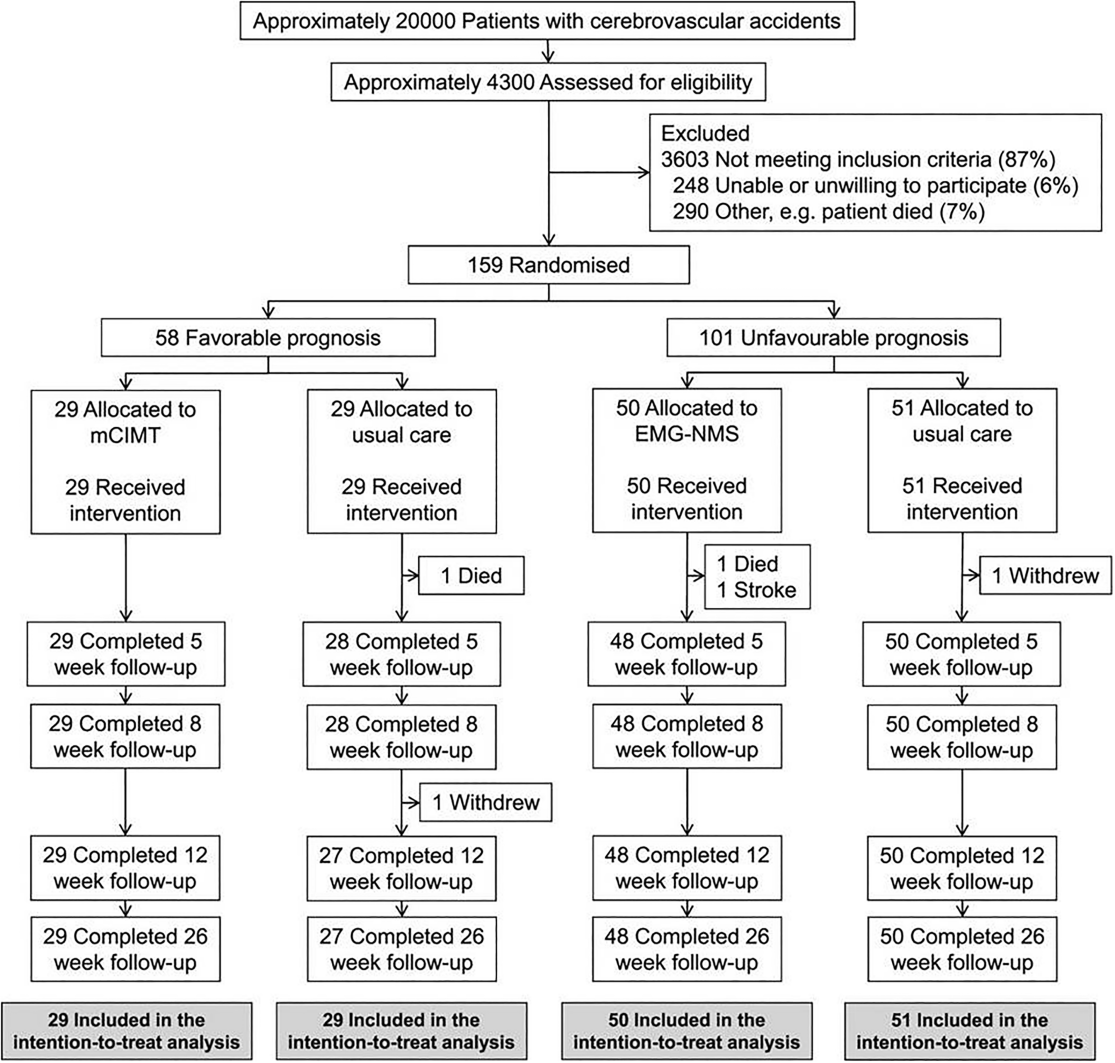
Flowchart of EXPLICIT-stroke Trial.

Age and gender-matched healthy control subjects were included when they (1) did not have a history of neurological and/or psychiatric disorders; (2) were aged between 18 and 80 years old; and (3) did not have metallic implants incompatible with the 3T MRI scanner. Informed consent for the trial was obtained in accordance with the declaration of Helsinki (2013) and the study protocol (S1 and S2 Files) was approved of by the local ethics committee (Medical Ethics Review Committees of Leiden University Medical Center (No. P08.035) and Dutch Central Committee on Research Involving Human Subjects (CCMO: No. NL21396.058.08)). The EXPLICIT-stroke Trial was registered in the Dutch Trial Registry (NTR, www.trialregister.nl, TC1424; see Fig 1). The authors confirm that all related trials for this intervention were registered.

### Clinical assessments

Patients’ baseline characteristics and neurological function were assessed within 2 weeks post-stroke onset, and included: age, gender, Bamford classification [22], Mini Mental State Examination [20], Cumulative Illness Rating Scale [23] and the modified Ranking Scale [24]. Overall neurological impairment was measured with the National Institutes of Health Stroke Scale [25], the degree of disability during activities of daily living with the Barthel Index [26] and motor strength of the upper-limb with the Motricity Index [27]. Patients’ synergistic motor control of the paretic arm was assessed at baseline (within 2 weeks post-stroke onset), 5 and 26 weeks post-stroke onset with the upper extremity section of the Fugl-Meyer Assessment (FMA-UE)[17].

### Scanning protocol

Of the 13 included patients, rs-fMRI data was collected at weeks 5 and 26 post-stroke onset. Images were acquired with two Philips Achieva 3.0 Tesla MR scanners (Philips, Eindhoven, the Netherlands), located at the University Medical Center Utrecht and Leiden University Medical Center.

For functional scanning, an EPI-pulse sequence was used with the following parameters: TR = 2200 ms, TE = 30 ms, flip angle = 80°, transverse orientation, FOV (AP, FH, LR) = 220 x 113 x 220 mm, slice gap = 0.272 mm. Slices were acquired in descending order. The acquired matrix had the following dimensions: 38 x 80 x 80, voxel size: 2.72 x 2.75 x 2.75 mm. The functional images where positioned to cover the entire cortex. 160 images were acquired during rs for a total duration of approximately 6 minutes. During scanning, patients were instructed to keep their eyes open and think of nothing in particular without falling asleep. The screen was blackened to keep visual input to a minimum.

High-resolution whole brain anatomical scans were acquired for all subjects as reference for functional activation maps (3D T1-weighted scan: TR = 9.717 ms; TE = 4.59 ms, flip angle = 8 degrees, 140 slices, 0.875 x 0.857 x 1.2 mm, FOV = 224 x168 x177 mm).

### Data pre-processing

Data were spatially pre-processed using Parametric Mapping (SPM12) software (http://www.fil.ion.ucl.ac.uk/spm/) in Matlab (Matlab 12; The Mathworks Inc, MathWorks, Natick, Massachusetts). The pre-processing entailed the realignment of all functional scans to the mean functional scan, slice time correction, and co-registration to the T1-weighted image. Normalisation to MNI (Montreal Neurological Institute) space was performed using SPM 12. Multimodal connectivity-based parcellation was included using the Brainnetome Atlas [28].

All subsequent analyses were performed using custom built routines in the Interactive Data Language (David Stern & ITT Visual Information Solutions, Boulder, Colorado, USA). For patients with lesions in the right hemisphere (n = 7), left and right ROI definitions were interchanged. Interchanging of left and right ROI definition was also done for an equal number of randomly picked control subjects to avoid a bias introduced by hemispheric asymmetries. Low frequencies were removed from the functional time series using a high-pass filter with a cut-off at 0.01 Hz. For each ROI the average time series was calculated and subsequently correlated with all other ROIs. The correlation coefficients (R) within the matrices were Fisher *Z* transformed for second level analysis, using $z^{\prime }=\left(\frac{1}{2}\right)\;\times \;ln\left(\frac{1+R}{1-R}\right)$.

### Statistical analysis

To evaluate time-dependent changes in rs-functional connectivity of motor networks, we calculated the mean connectivity between the ROIs comprising the motor system (average Z-score for all pairs of Caudal Middle Frontal, Paracentral, Postcentral, Precentral) within the ipsilesional and contralesional hemisphere, for the first and second session (week 5 and 26 post-stroke onset). This resulted in two connectivity values for each session, one for the ipsilesional, and one for the contralesional hemisphere. The same connectivity values were calculated for the single session of the healthy control subjects.

To evaluate time-dependent changes in the motor network of patient, we performed a 2 x 2 repeated measures ANOVA with session (week 5 versus 26) and hemisphere (affected versus non-affected) as within subject factors. In addition, we investigated the relation between magnitude of time-dependent changes in rs-functional connectivity and time-dependent changes in motor impairment as measured with the FMA-UE. The change in FMA-UE between week 5 and week 26 was tested using a paired-samples t-test (two-sided). To investigate rs-functional connectivity differences per hemisphere between patients and control subjects, we compared rs-functional connectivity of the patients at week 5 post-stroke with the rs-functional connectivity of control subjects using a repeated measures ANOVA, with hemisphere (ipsilesional/contralesional) as within subjects-factor, and group (patient/control) as between subjects-factor.

To further investigate potential changes in connectivity beyond the hypothesized areas, we performed an additional analysis. We tested differences in the connections between all ROI pairs between week 5 and week 26 after stroke using paired-samples t-tests, and between patients at week 5 and control subjects using paired samples t-tests (p <.05 with Bonferroni corrections for the number of tests, n = 29890: p <.00000167). Additionally, to measure overall effects instead of focussing on every corrected significant ROI, we also observed the actual proportion of significant tests while keeping the threshold at p <.05.

## Results

The characteristics of the patients included in this study are presented in Table 1. The average time post-stroke onset at which the first and second rs-fMRI took place was 35.6 days (SD = 4.4) and 187.0 days (SD = 6.4) respectively. From 35.6 days (week 5) to 187.0 days (week 26), patients showed an average improvement in upper-limb function of 4 points (FMA-UE; Z = -2.32, p =.020 (two-tailed).

**Table 1:** Demographic and stroke characteristics measured at 8 days, 35.6 days, and 187 days post-stroke.

	Patients (n = 13)	Healthy Controls (n = 13)
*Baseline*		
Age (years (SD))	63.6 (9.0)	55.1 (9.0)
Gender (% male)	84.6	76.9
Bamford (LACI/PACI/TACI; %)	69.2/15.4/15.4	NA
MMSE (0–30)	27.2 (SD: 3.1; IQR: 25.3–30.0)	30
CIRS (0–52)	2.3 (SD: 1.5; IQR: 2.0–3.0)	0
NIHSS Total (0–42)	5.8 (SD: 2.6; IQR: 4.0–7.5)	0
Modified Ranking Scale (0–5)	3.9 (SD: 0.7; IQR: 3.0–4.0)	0
Barthel Index (0–20)	10.1 (SD: 5.1; IQR: 7.5–14.5)	20
Motricity Index arm (0–100)	50.3 (SD: 28.9; IQR: 34.0–74.0)	100
FMA-UE (0–66)	29.6 (SD: 18.5; IQR: 11.0–46.0)	66
Time post-stroke onset (days)	7.2 (2.5)	NA
*Week 5 after stroke*		
Time post-stroke onset (days)	35.6 (4.4)	NA
Barthel Index (0–20)	18.2 (SD: 2.7; IQR: 17.5–20.0)	20
Motricity Index arm (0–100)	81.5 (SD: 16.3; IQR: 76.0–96.0)	100
FMA-UE (0–66)	53.5 (SD:14.1; IQR: 47.0–63.0)	66
*Week 26 after stroke*		
Time post-stroke onset (days)	187.0 (6.4)	NA
Barthel Index (0–20)	19.9 (SD: 0.3; IQR: 20.0–20.0)	20
Motricity Index arm (0–100)	88.1 (SD: 10.6; IQR: 84.0–100.0)	100
FMA-UE (0–66)	60.9 (SD: 3.95; IQR: 57.5–65.0)	66

F: female; M: male; MMSE: Mini Mental State Examination; CIRS: Cumulative Illness Rating Scale; NIHSS: National Institutes of Health Stroke Scale; FMA-UE: Fugl-Meyer Assessment of the Upper Extremity; IQR: Inter Quartile Range; NA: Not Applicable, i.e. healthy control subjects did not have neurological or upper-limb impairments.

Three patients had infarctions extending to cortical areas, whereas 10 patients had subcortical infarctions (Table 2). In more detail, 9 lesions were located in the basal ganglia, 2 in the pons, and 1 of the patients had an infarction involving the primary motor cortex (Brodmann area 4).

**Table 2:** Subcortical and cortical areas (including hemisphere of stroke) containing the lesion for individual patients.

Patient	Subcortical area (hemisphere)	Cortical area (hemisphere)
**1**	Putamen (left)	NA
**2**	Putamen (left)	NA
**3**	Caudate (left)	NA
**4**	Caudate (left), Putamen (left)	NA
**5**	Brainstem	NA
**6**	Putamen (right)	NA
**7**	Thalamus (left), Putamen (left)	NA
**8**	NA	Postcentral (right), Supramarginal (right), Parsopercularis (right), Inferior parietal (right)
**9**	NA	Postcentral (right), Supramarginal (right)
**10**	Caudate (right)	NA
**11**	Caudate (right)	Precentral (right)
**12**	Putamen (right)	NA
**13**	Brainstem	NA

NA: Not Applicable

Time-dependent differences in rs-functional connectivity of motor networks in the contralesional and ipsilesional hemisphere: A main effect of hemisphere (affected versus non-affected) was observed (F(1,12) = 4.838, p = .048), indicating that the rs-functional connectivity of the ipsilesional hemisphere (average = .465, SD = .056) was lower than the rs-functional connectivity of the contralesional hemisphere (average = .511, SD .049). No main effect of session (week 5 versus week 26) was observed (F(1,12) = .667, p = .430), indicating that rs-functional connectivity in week 5 (average = .474, SD = .053) was comparable to week 26 (average = .502, SD = .055). Additionally, no interaction between hemisphere and session was found (F(1,12) = .618, p = .447), indicating that hemisphere differences in rs-functional connectivity were comparable over time (Table 3).

**Table 3:** Rs-functional connectivity scores of the affected and non-affected hemisphere, split for time post-stroke onset (weeks 5 and 26).

Patient	Affected hemisphere week 5	Unaffected hemisphere week 5	Affected hemisphere week 26	Unaffected hemisphere week 26
1	.93	.92	.97	.98
2	.41	.52	.55	.69
3	.74	.66	.62	.61
4	.27	.61	.29	.60
5	.23	.33	.41	.37
6	.48	.55	.64	.43
7	.53	.50	.50	.52
8	.14	.13	.23	.21
9	.31	.26	.50	.41
10	.40	.58	.45	.61
11	.36	.29	.36	.48
12	.41	.58	.54	.61
13	.56	.58	.50	.57

Relation between magnitude of time-dependent changes in rs-functional connectivity and upper-limb motor recovery (FMA-UE) in stroke patients: Even though the previous analysis did not provide evidence that the difference in rs-functional connectivity of the ipsilesional hemisphere and the contralesional hemisphere changed over time at group level, individual differences in time-dependent changes could still be present and related to upper-limb motor recovery. No relations, however, were observed between time-dependent changes in the difference in rs-functional connectivity between the affected and non-affected hemisphere and upper-limb motor recovery (Spearman's rho coefficient .342, p = .253 (two-tailed)).

Potential differences in rs-functional connectivity between stroke patients (at 5 weeks post-stroke onset) and healthy control subjects: No overall group effect was observed (F(1,24) = 3.020, p = .095). No significant interaction between hemisphere (affected versus non-affected) and group (stroke patients versus healthy control subjects) was obtained (F(1,24) = 3.303, p = .082) (Table 4).

**Table 4:** Rs-functional connectivity scores for affected versus non-affected hemispheres in stroke patients at 5 weeks post-stroke onset, compared to rs-functional connectivity per hemisphere of reference in healthy control subjects.

Affected hemisphere week 5	Unaffected hemisphere week 5	Healthy controls hemisphere of reference	Healthy controls hemisphere of reference
.93	.92	.78	.82
.41	.52	.65	.63
.74	.66	1.03	.99
.27	.61	.39	.46
.23	.33	.31	.23
.48	.55	.59	.72
.53	.50	.34	.35
.14	.13	.68	.62
.31	.26	.41	.48
.40	.58	.78	.78
.36	.29	.89	.88
.41	.58	.42	.40
.56	.58	.70	.69

**Fig 2:**
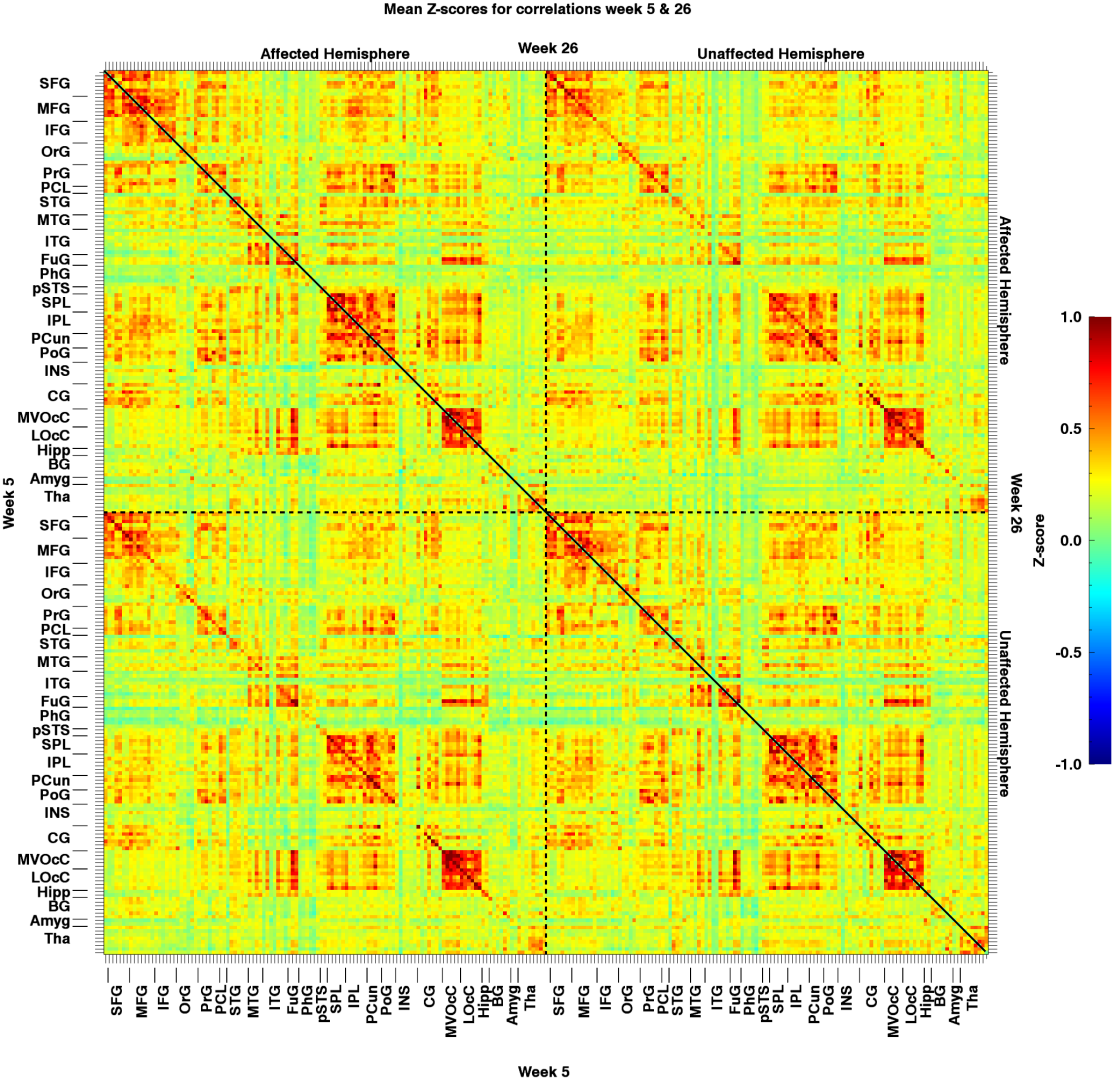
Mean resting-state Z-transformed correlations of the patients (n = 13) between all regions of interest. The matrix area on the bottom left of the diagonal represents the correlations for week 5, while the area on the top right represents the correlations for week 26. Note that the colour representation of the values was clipped beyond -1 and 1, although due to the Fisher transformation values can actually exceed the [-1,1] interval. AH: Affected Hemisphere; UA: Unaffected Hemisphere; lesion location = left hemisphere.

**Fig 3:**
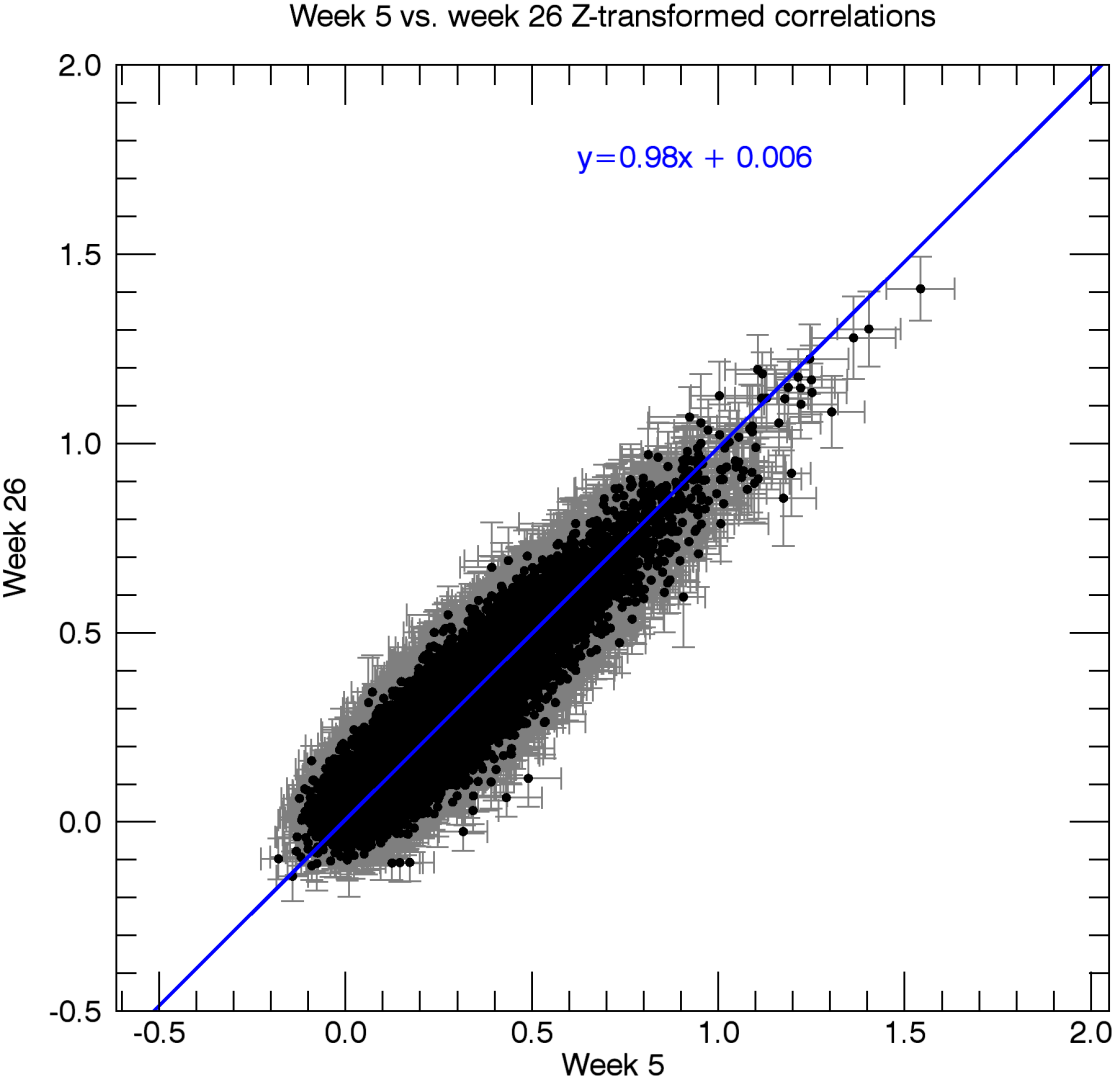
Scatterplot of the resting-state correlations in the patients for week 5 vs. week 26. Every data point in the graph represents an ROI pair, with the x-coordinate indicating the mean Z-score (n = 13) for the correlation between the two ROIs at week 5, and the y-coordinate indicating the mean Z-score between the two ROIs at week 26. Grey bars indicate the standard error of the mean for week 5 (horizontal bar) and week 26 (vertical bar). The blue line depicts a straight line fit through the data points [29].

**Fig 4:**
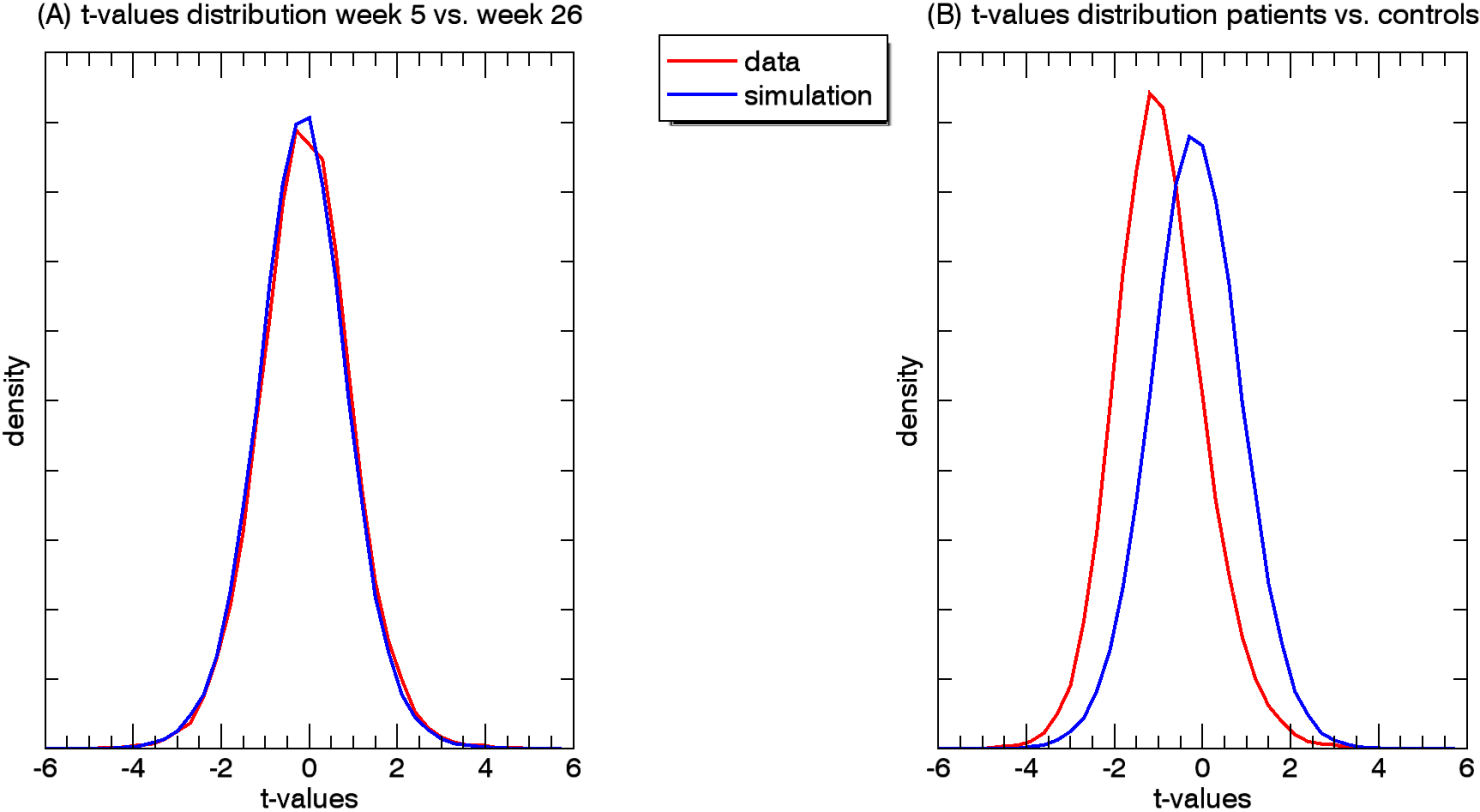
A. Density of t-value distributions for the patients for week 5 versus week 6. B. Density of t-value distributions for the patients versus healthy controls. For both comparisons, the distributions are largely comparable. The simulation distribution reflects the null hypothesis distribution.

Potential changes in rs-functional connectivity within the motor networks in stroke patients: When testing for additional effects beyond the hypothesized connection, we observed no differences in connectivity between week 5 and week 26, regarding all 29890 ROI-pairs (t_crit_ = 8.11; t_max_ = 4.97 and t_min_ = -5.83). In other words, this analysis revealed no changes in rs-functional connectivity from 5 to 26 weeks post-stroke onset (Figs 2, 3 and 4A).

**Fig 5:**
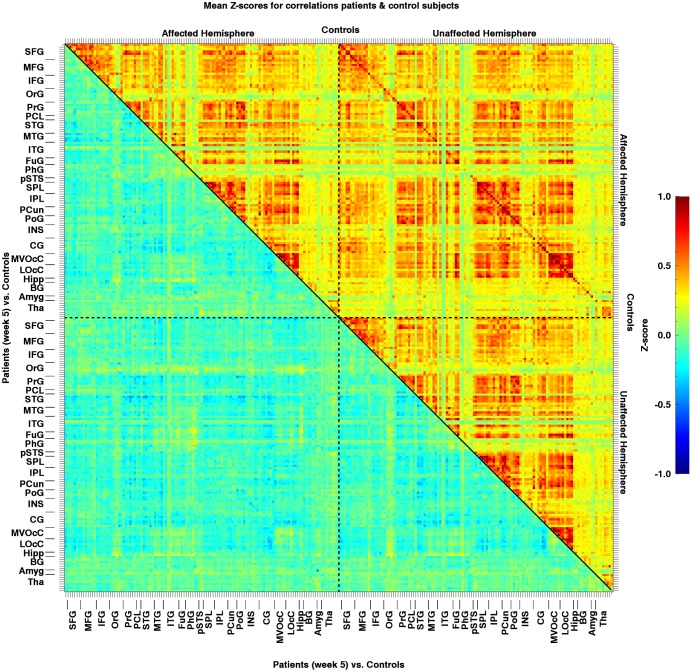
Mean resting-state Z-transformed correlations for patients at week 5 (n = 13) vs. control subjects (n = 13) between all regions of interest. The matrix area on the top right of the diagonal represents the correlations for control subjects, while the area on bottom left represents the difference in correlations between patients at week 5 and healthy controls. The colour representation of the values was clipped beyond -1 and 1.

**Fig 6:**
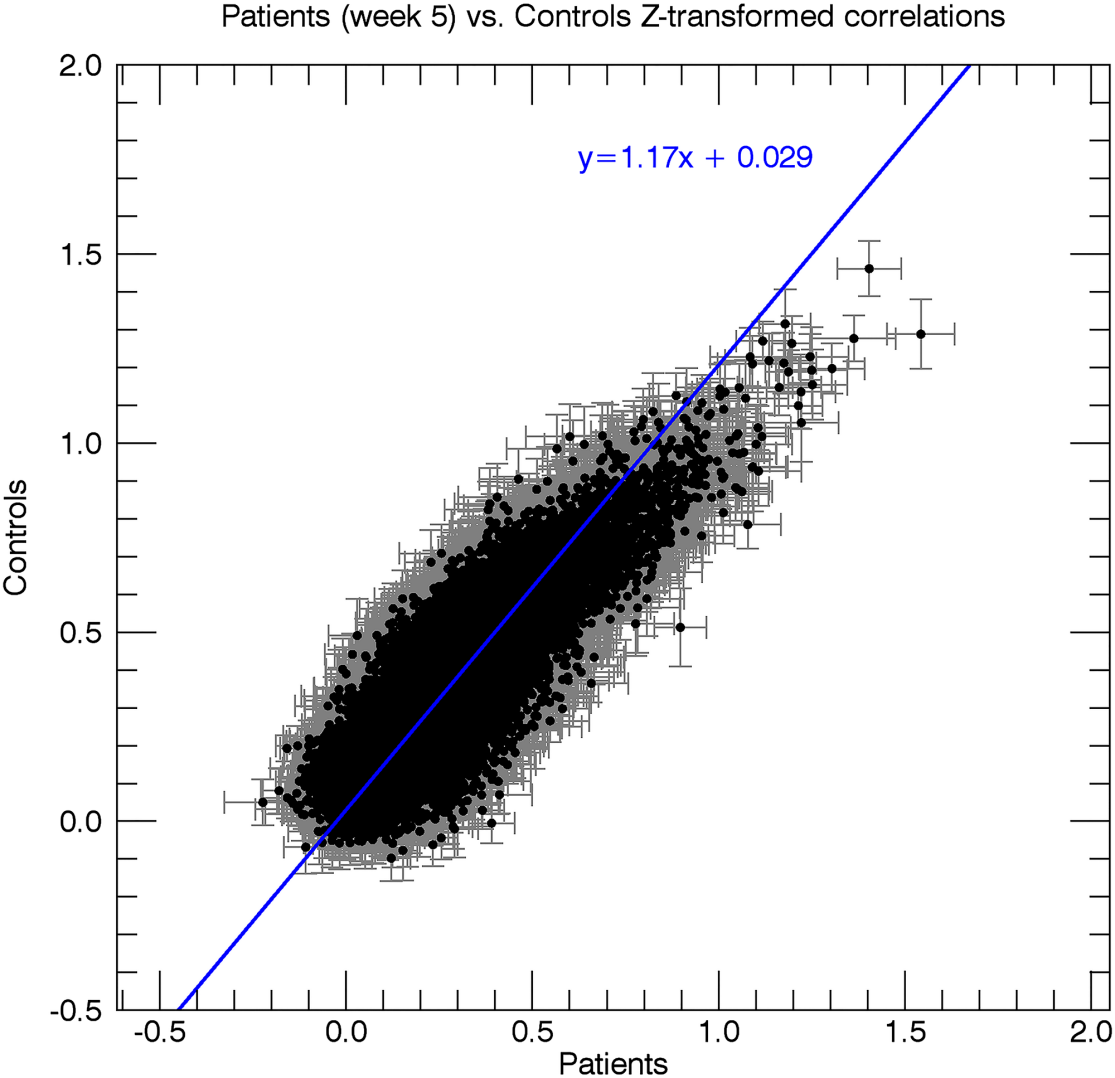
Scatterplot of the resting-state correlations in the patients at week 5 vs. healthy controls. Every data point in the graph represents an ROI pair, with the x-coordinate indicating the mean Z-score (n = 12) for the correlation between the two ROIs for patients at week 5, and the y-coordinate indicating the mean Z-score between the two ROIs for control subjects. Grey bars indicate the standard error of the mean for patients at week 5 (horizontal bar) and healthy controls (vertical bar). The blue line depicts a straight line fit through the data points.

When comparing all connections between patients (week 5 post-stroke) and healthy controls, we found that a total of 4 out of the 29890 connections were significantly decreased in patients. None of the 29890 connections showed a significant increase compared to healthy controls (Figs 4B, 5 and 6). When comparing all connections between week 5 and week 26 post-stroke, 1362 of the 29890 correlations (4.6%) were below threshold (p < .05), and thus significant. At first sight, this might seem surprising, as 4.6% is less than would be expected by chance alone. It is, however, unlikely that these results reflect a pivotal underlying phenomenon, as the elements in the correlation matrices are unlikely to represent truly independent measurements. For example, the number of time series underlying a correlation matrix is only equal to the number of ROIs, and changes in a single time series affect an entire row/column in the matrix. In addition, brain networks may include several ROIs, which further enhances dependency. These dependencies across matrix elements increase the volatility of correlation values averaged across multiple elements as opposed to when the elements would be completely independent. The 'significantly' fewer than expected correlations reaching the 5% threshold most likely reflects this phenomenon.

## Discussion

The aims of the present study were fourfold: to investigate (1) time-dependent changes in rs-functional connectivity of motor networks in stroke patients; (2) the relation between magnitude of time-dependent changes in rs-functional connectivity and time-dependent changes in motor impairment as measured with the FMA-UE in stroke patients; (3) potential differences in rs-functional connectivity between stroke patients (at 5 weeks post-stroke onset) and healthy control subjects; and (4) potential changes in connectivity outside the motor system in stroke patients.

In stroke patients, the ipsilesional rs-functional connectivity between motor areas was lower compared to the contralesional rs-functional connectivity, but this difference did not change over time. No relations were observed between individual changes in rs-functional connectivity and upper-limb motor recovery, despite changes in upper-limb function as measured with the FMA-UE. Last, overall rs-functional connectivity was comparable for patients and age- and gender matched healthy control subjects.

There are a few possibilities for the observed discrepancy between changes in upper-limb motor recovery and the lack of significant changes in rs-functional connectivity. First, the improvement over time was 4 points on the FMA-UE, which is a 6% change of the maximum possible score of 66 points over time. Such a percentage is often considered not to be clinically relevant [30–32], stressing the importance of caution on interpreting these effects at the behavioural level. Second, spontaneous neurobiological recovery as reflected by improvements in FMA-UE scores [33], is mainly restricted to the first 5 weeks post-stroke in patients with moderate to mild upper-limb paresis [34]. We assume that structural changes leading to improvements in rs-functional connectivity occur in the same time window. Therefore, these structural changes may have occurred prior to the first rs-fMRI measurement in the present study. [13–16]. The lack of significant changes in rs-functional connectivity is also in line with our longitudinal kinematic studies in which we found that improvements in intra-limb coordination dynamics are mainly restricted to the first 5 weeks post-stroke, whereas minor improvements are found beyond this time window of spontaneous neurobiological recovery [35, 36].

For further interpretation of our results, it is important to realise that previous studies that showed large significant changes in functional connectivity were mainly based on populations of *severely affected* patients (Xu, Qin, Chen, Jiang, Li, Yu, 2014). Our patient population presented with mild upper-limb impairments and could therefore only show a limited amount of improvement (i.e. ceiling effect). The changes in brain activation patterns (i.e. cerebral reorganization) may therefore have been smaller in our patient population in comparison to a population of severely impaired patients. Furthermore, most studies reporting significant correlations between cortical reorganization and functional upper-limb recovery used task-related brain activation during active motor tasks (Ward Brain 2013). One might argue that task-related activity represents a more direct measure for identifying changes in brain activity patters in relation to upper-limb recovery in mild to moderately impaired patients. However, as task performance is more difficult to control for as compared to compliance with rs, longitudinal changes in task-related activity can be confounded by differences in quality of motor performance while executing a task. Another study in this cohort indeed demonstrated that motor abnormalities during grasping are subtle, with patients compensating for the deficit instead of actually showing true neurological recovery of neurological impairments (Buma et al., 2016). In other words, changes in motor task fMRI might thus reflect behavioural compensatory mechanisms rather than behavioural restitution of neuronal deficits.

We found hemisphere specific abnormalities; the connectivity within the affected hemisphere was lower when compared to the unaffected homologue at 5 weeks post-stroke. This difference between the affected area and the unaffected homologue did not change over time. Additionally, comparing patients at 5 weeks post-stroke with age and gender-matched healthy control subjects revealed only 4 significantly decreased correlations in the patient group out of the 29890 correlations.

The present study had some limitations. First, the sample of stroke patients was relatively small. This most likely has had an effect on the power needed to find potential differences. Second, our moderately impaired stroke patients were required to have some remaining hand function (i.e. still be able to perform voluntary flexion and extension of the fingers of the paretic upper-limb) for the task-fMRI subproject of the EXPLICIT-stroke Trial, so current results may not be generalizable to patients with more severe stroke [18]. Despite these relatively mild impairments, the abnormality in ipsilesional connectivity was significant. Third, the cerebellum has been found to play an important role in the compensation of motor impairment. In our sample of patients, however, the cerebellum was *not* included in the MRI scans in *all* patients. Therefore, we were unable to include the cerebellum in our analyses. Fourth—and somewhat related to the third limitation—, different motor areas are responsible for distinct motor functions. Here, averaged rs-functional connectivity within the motor network of one hemisphere, between hemispheres, and between the motor network and other networks were analysed. However, the motor areas were not checked separately (see Carter, et al, 2009; Siegel, et al, 2016). Fifth, an additional shortcoming is the relatively small magnitude of upper-limb recovery between week 5 and week 26 post-stroke. The changes in FMA-UE was largest in between week 1 and 5 (28 points on average), but no rs-fMRI data were collected at week 1. The observed changes in fMRI connectivity may thus not represent the full range of post-stroke changes. Importantly, although the improvement between week 5 and 26 was mathematically significant, it was not regarded as clinically significant. Last, the disturbance in rs-activity early after stroke may reflect functional dysfunction caused by interhemispheric diaschisis [37, 38]. Xu and co-workers [16] found a decreased interhemispheric functional connectivity between the ipsilesional and contralesional primary sensorimotor cortex *early* after stroke which increased to near normal levels after 3 months post-stroke onset. In our sample, potential large scale disturbances in rs-activity after stroke were not present at 5 weeks after stroke and as stated above, we did not measure rs-activity earlier than 5 weeks post-stroke onset.

To investigate the differences in cerebral reorganization between subgroups, future studies should include a larger population of stroke patients, including large enough subgroups of patients with various degrees of functional motor impairment. As most spontaneous neurobiological recovery occurs within the first 8–10 weeks post-stroke (Kwakkel 2006), and is assumed to be the main driver for recovery of structural connectivity early post stroke, we recommend that rs-fMRI measurements should preferably start within the first weeks post stroke, with frequent follow-up measurements at fixed time-points post-stroke onset, at least up to 3 months after stroke.

To conclude, the current findings did not provide evidence that in moderately impaired stroke patients the lower rs-functional connectivity of the ipsilesional hemisphere changes over time. Yet the sample size limits the firm final conclusions.

## Supporting information

S1 File Explicit protocol. (DOC)

S2 File CONSORT 2010 checklist. (DOC)
