# Supplementary material for: No changes in functional connectivity during motor recovery beyond 5 weeks after stroke; A longitudinal resting-state fMRI study
Source: PLoS One. 2017 Jun 8;12(6):e0178017. doi: 10.1371/journal.pone.0178017 (PMC5464555; doi:10.1371/journal.pone.0178017)
Supplement: S1 File — (DOC) [file pone.0178017.s001.doc]

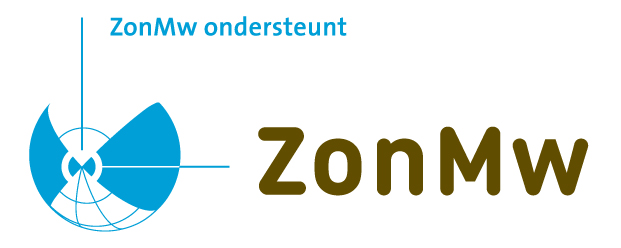


**[
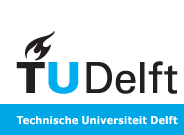
](http://www.tudelft.nl/)
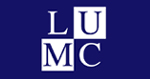

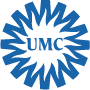

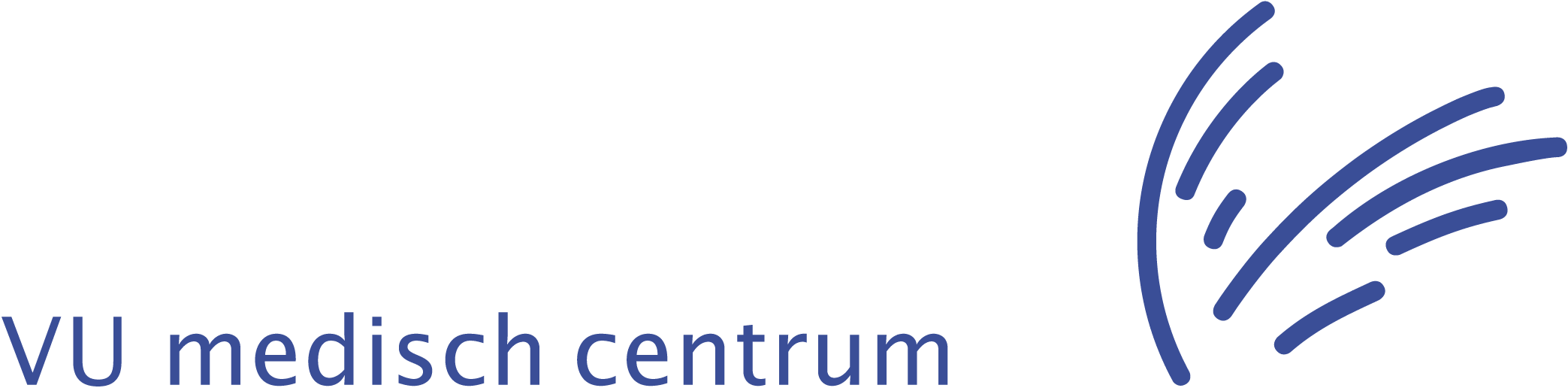

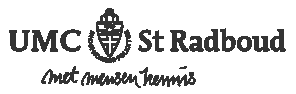
**

##

**EXPLAINING PLASTICITY AFTER STROKE (EXPLICIT- STROKE)**

*A multicenter research program consisting of a randomized clinical trial on the effect of early intervention in stroke rehabilitation and a longitudinal survey into the dynamics of post-stroke recovery.*

*EXPLICIT has been funded by ZON-MW in the framework of the 2^nd^ Rehabilitation program.*

**Principal Investigators:**

**Leiden University Medical Centre:**

Drs. J.M. van der Krogt, Dr. C.G.M. Meskers, Dr Ir. J.H. de Groot, Dr S.A.R.B. Rombouts, Prof. Dr M.A. van Buchem, Prof. dr. J.G. van Dijk, Prof. dr. J.H. Arendzen.

**VU Medical Centre:**

J van Kordelaar, Dr. E. van Wegen, Prof. dr. P.J. Beek, Dr. G. Kwakkel.

**UMC St. Radboud**

Drs. C. Bakker, Dr. A.A. van Kuijk, Dr. H.J. Hendricks, Dr. J.W. Pasman, Prof. dr. M.J. Zwarts, Prof. dr. A.C. Geurts

**UMC Utrecht**

F. Buma, Dr. G. Kwakkel, Dr. N. Ramsey, Dr. L.J. Kapelle, Dr. V.P.M. Schepers, Prof dr. E. Lindeman.

**TU Delft**

OIO (vacancy), Dr. Ir. A.C. Schouten, Dr. Ir. E. de Vlugt, Prof. dr. F.C.T. van der Helm

#
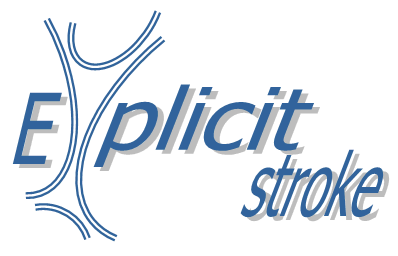


**Contact information:**

Drs J.M. van der Krogt

Leiden University Medical Centre

Department of Rehabilitation Medicine, B0Q

PO Box 9600

2300 RC Leiden, The Netherlands

Tel. 0031-71-5263457

Fax. 0031-71-5266697

**Contents**

[Summary 3](#__RefHeading___Toc188111260)

[1. Introduction 4](#__RefHeading___Toc188111261)

[2. Study design 5](#__RefHeading___Toc188111262)

[3. Clinical outcome variables 8](#__RefHeading___Toc188111263)

[4. Patients and Methods 10](#__RefHeading___Toc188111264)

[Project A: early intervention 11](#__RefHeading___Toc188111265)

[Project B: cortical reorganisation and compensation strategies 11](#__RefHeading___Toc188111266)

[Project C: longitudinal relations 11](#__RefHeading___Toc188111267)

[5. Statistical analysis 13](#__RefHeading___Toc188111268)

[6. Time schedule for the project 14](#__RefHeading___Toc188111269)

[7. Financial data 15](#__RefHeading___Toc188111270)

[8. Knowledge transfer 15](#__RefHeading___Toc188111271)

[9. Literature 15](#__RefHeading___Toc188111272)

[10. Appendices **Fout! Bladwijzer niet gedefinieerd.**](#__RefHeading___Toc188111273)

# Summary

Prospective cohort studies show that about 80% of all stroke survivors have an upper limb paresis immediately after stroke. Only one third of all stroke patients will regain some dexterity, whereas well-researched evidence based therapies for an effective treatment of the upper limb are lacking.

However, the main claim of the literature is that functional recovery of the upper paretic limb is mainly defined within the first month post stroke and that rehabilitation services should be applied preferably within this time window of recovery. Furthermore, it is known that exercise-related interventions are most effective when they are applied intensively in a task-oriented way. The program EXplaining PLastICITy after stroke (acronym: EXPLICIT-Stroke) builds on the existing knowledge about functional prognosis and effectiveness of exercise therapy in patients with a first-ever MCA stroke.

The two RCT’s of EXPLICIT are aimed to investigate the effects of early intensive intervention, i.e. 1) early applied Constraint Induced Movement Therapy (CIMT) in patients with a known favourable prognosis for functional recovery and 2) early started sensorimotor stimulation program by Electromyography-triggered neuromuscular electrical stimulation (EMG-NMS) in patients with a poor probability for functional recovery of the upper limb. In both single-blinded RCT's, interventions will be compared to usual practice (standard rehabilitation program).

To improve our knowledge on what patients learn when they show functional recovery, EXPLICIT will explore the underlying mechanisms that are involved in regaining dexterity. For this latter purpose, a longitudinal study is performed on the impact of intactness of the primary motor system as revealed with TMS to be related to: 1) changes in cortical activation patterns of ipsi- and contralateral brain by fMRI; 2) adaptive motor control of the upper limb (kinematics) and 3) changed stiffness of the upper paretic limb by haptic robotics in a repeated measurement design.

By this, EXPLICIT will provide an answer to the key question whether therapy induced improvements are due to either a reduction of basic motor impairment by neural repair (i.e., restitution of function) and/or the use of behavioral compensation strategies (i.e. substitution of function).

# 1. Introduction

EXPLICIT: Explaining Plasticity after stroke, is a Dutch study researching the possibilities of regaining arm and hand function in the acute and sub acute phase post stroke. It is a multi-centre study, with participation of the LUMC, VUMC, UMC Utrecht and Radboud UMC, including their affiliated hospitals and rehabilitation centres.

Each year, more than 32,000 patients in the Netherlands sustain a stroke (Loor et al, 1999) and the incidence is expected to have increased by 30–45% in 2015 (Ruwaard, 1997). About 80% of the survivors have an upper limb paresis immediately after stroke (Nakayama, 1994), whereas only one third of all stroke patients have regained some dexterity at 6 months (Dobkin et al, 2005). Recent prospective cohort studies showed that functional recovery (regaining dexterity) at 6 months post-stroke is highly predictable within the first 4 weeks (Kwakkel et al, 2003; Dobkin et al, 2005).

Figure 1: Probability of regaining dexterity based on early Action Research Arm Test (ARAT). A high ARAT score

predicts a high probability for regaining dexterity (red line) a low score indicates a low probability of regaining

dexterity (green line)

The underlying mechanisms responsible for recovery are, however, not well understood. (Nudo et al, 2001; Kwakkel et al, 1999; 2004b). A better understanding of the factors that facilitate upper limb recovery, as well as the time windows in which these recovery mechanisms work best are a prerequisite for improving our rehabilitation services in the future.

A number of studies suggest that rehabilitation services should be initiated as soon as possible, preferably within the critical time window of 4 weeks post stroke (Kwakkel et al, 2003).

In addition, the treatment should be applied intensively and should take a task-oriented form (Kwakkel et al, 1999; 2004a & 2004b; Dobkin et al, 2005).

Recently, several controlled trials in the chronic phase after stroke have shown that innovative therapies such as constraint induced movement therapy (CIMT) (van der Lee et al, 1999; Wolf et al, 2006), as well as certain forms of EMG-triggered neuromuscular electrical stimulation (EMG-NMS) (Bolton et al, 2004), may improve upper limb function.

A few studies have found that improvements induced by CIMT (Dijkhuizen et al, 2001; Schaechter et al, 2003) or EMG-NMS of the affected upper limb (Stephan et al, 1997; Kimberley et al, 2004) coincide with cortical reorganizations of motor maps in patients suffering from chronic stroke. This suggests that neuroplasticity is an important mechanism for functional recovery of the upper paretic limb. However, the impact of the above-mentioned therapies on dynamics of cortical reorganization has never been investigated in patients with (sub)acute stroke.

Recent longitudinal studies also suggest that functional recovery is more than neural repair alone. Repeated measurements in time show that improved reaching and grasping activities with the paretic upper limb depends also on adaptive trunk movements (Cirstea et al, 2003; Kwakkel et al, 2004b).

Therefore, understanding functional recovery of the upper limb requires not only knowledge about longitudinal changes in neuroplasticity but also knowledge about the compensation strategies patients use and the changing biomechanics in motor control as a function of time

The functional impact of the time-dependent changes in cortical neuroplasticity, as well as the adaptive compensation strategies used to deal with existing motor deficits and biomechanical constraints, need to be explored in order to improve our knowledge about what exactly patients learn when functional improvement is observed.

This study aims to get a better understanding of the neurobiological background of functional recovery of the upper limb after stroke, especially focussing on early intervention.

The results of EXPLICIT will allow clinicians to define both the optimal treatment strategy and its time window in rehabilitation in the near future.

The clinical outcome measures may serve as a template for understanding functional recovery by elucidating the longitudinal relationship between the body functions, body structures, activities and participation (as described in the International Classification of Functioning (ICF). The EXPLICIT-study is designed to answer the following questions: Is early intervention with CIMT or EMG-NMS effective compared to our current rehabilitation programme? How are therapy-induced improvements in upper limb function after stroke longitudinally related with observed changes in (sub)cortical reorganisation and using compensation strategies?

# 2. Study design

As mentioned above, the study will mainly focus on two key areas in stroke rehabilitation: 1) effectiveness of early applied, intensive task-oriented intervention and 2) underlying mechanisms of functional repair in terms of cortical reorganisation versus using adaptive compensation strategies. This leads to a research program, divided into three projects, which are highly interlinked :

*2.1 Project A: early intervention. Objective: two single blinded randomized controlled trials investigating the effectiveness of early interventions on paretic limb recovery: Constraint Induced Movement Therapy (CIMT)(A1) and EMG-triggered Neuromuscular Stimulation (EMG-NMS)(A2) versus conventional therapy.*

A1. Constraint induced movement therapy (CIMT)

The claim made in the literature that therapy should be intensive and task-oriented, as well as the finding that therapy is most effective in the early stages after stroke, both represent basic assumptions in the EXPLICIT research program. So far, it is assumed that the impairment of hand function is exacerbated by learned non-use and that this in turn leads to a loss of cortical representation of the upper limb (Sunderland, 2005). There is increasing evidence that these processes can be reversed by constraining the unaffected limb, combined with intensive practice of the paretic hand (i.e. shaping procedures). In the subacute phase after stroke some evidence exists that CIMT increases spontaneous use of the hand by recovery of existing deficits, either through reduction of learned non-use or by overcoming the sense of effort during movement (Sunderland, 2005).

A2. Electromyography-triggered neuromuscular electrical stimulation (EMG-NMS)

The assumed positive impact of EMG-NMS is based on the finding that 2 hours of sensory stimulation of the median nerve of a paretic limb may result in improved pinch strength (Conforto et al, 2002). This improvement in muscle strength correlates with the stimulus intensity and has been identified in the absence of motor training. Virtual imaging has also been found to improve cortical plasticity and motor performance of the upper limb in chronic stroke (Stephan et al, 1997; Kimberley et al, 2004). The above results suggest that somatosensory stimulation, combined with virtual imaging by applying EMG-NMS, may be a promising adjuvant therapy to initiate active motor control in stroke patients with an upper limb deficit. It is especially early after stroke, when the outcome in terms of dexterity has not yet been defined, that EMG-triggered feedback (EMG-NMS) of wrist and finger extensors are hypothesized to facilitate cortical ipsi-lesional and contralesional plasticity and hence increase the probability of return of some functional dexterity (Stephan et al, 1997; Kimberley et al, 2004). In addition, it is believed that the movement imagery component causes neural networks in the motor cortex, associated with the specific imagined movement, to be stimulated and reinforced. Evidence supporting this theory has been found in fMRI-based studies (Stephan et al, 1997; Kimberley et al, 2004).

Project A1: CMIT

*Research questions:*

1. Is an early intensive physical/occupational therapy programme lasting 30 minutes, followed by 3 hours of CIMT for 5 consecutive working days over a period 3 weeks more effective in terms of recovery of motor function and dexterity of the paretic limb than a conventional treatment programme in patients suffering from a first-ever MCA stroke with an initially favourable prognosis of the upper limb?
2. Are CIMT-induced improvements in dexterity sustained up to 6 months post stroke when compared to improvements in patients who receive the conventional exercise programme?

Project A2: EMG-NMS

*Research question:*

1. Is an early EMG-NMS programme of the wrist and finger extensors lasting 30 minutes per working day for 3 weeks more effective in terms of the probability of return of dexterity at 6 months than a conventional treatment programme over a period of 3 weeks?

*2.2 Project B: cortical reorganisation and compensation strategies. Objective: translational research investigating the predictive value of Transcranial Magnetic Stimulation (TMS)(B1), the clinical significance of changing signal activity in functional MRI (B1), the role of compensation strategies (kinematics)(B2) and how changes in stiffness and spinal reflex properties interfere with upper limb recovery (haptic robot) (B3)*

No studies have so far analysed in detail whether improvement in dexterity reflects reduction of basic motor impairments or works by learning compensatory movement strategies. To date, there is a growing weight of evidence in favour of compensatory learning strategies (Kwakkel et al, 2004; Sunderland, 2005). For example, there are strong indications that motor recovery after cortical injury occurs to a large extent through behavioural compensation strategies, rather than via processes of true recovery alone (Kwakkel et al, 2004b; Sunderland, 2005; Geurts et al, 2005). Theoretically, behavioural compensation strategies in which deficits are taken over by motor control of the less-affected trunk are considered to be potential confounders in our understanding of ‘neural repair’ and related cerebral recovery after stroke (Kwakkel et al, 2004; Sunderland, 2005). For example, in one recent study of stroke patients, moderately to severely impaired subjects used compensatory strategies of the trunk to accomplish a pointing task, rather than trying to achieve restitution of the original arm function (Cirstea et al, 2000, 2003). Cortical changes detected by fMRI may therefore reflect alternative (compensatory) motor skill learning rather than restitution of stroke induced impairments. As a result, these studies fail to differentiate between ‘motor recovery based on true repair’ and ‘motor recovery based on using compensation strategies’. This has caused considerable difficulty in the interpretation of such changes in fMRI studies, apart from other potential confounding factors such as a lack of control over mirror movements, strength, precision and the timing of imaginary movements (motor paradigm) during fMRI scanning. Finally, understanding the observed adaptive behavioural movement strategies requires that the biomechanical changes of the musculoskeletal system are measured longitudinally. For this purpose, it is of vital importance to understand the relationship between parameters characterizing CNS function (such as TMS and fMRI) and parameters describing end-point mechanical behaviour and peripheral reflex-chain properties.

Project B1: fMRI/TMS

*Research questions:*

1. Are CIMT-induced gains reinforced by recruitment of cortical activation in the ipsi-lesional hemisphere compared to patients receiving usual care?
2. Is it possible to explain the recovery of dexterity in the paretic extremity during the first 6 months post stroke from the changes in cortical activation found in the first 4 weeks on fMRI in M1, vPMA, dPMA and SMA cortices of infarcted and non-infarcted cerebral hemispheres in patients with primary ischemic middle cerebral artery stroke?
3. Are changes in fMRI patterns in the contra-lesional cortex (M1) related to the impact of cortico-spinal tract degeneration as established with TMS within the first week and at 6 months post stroke?
4. What is the predictive value of TMS parameters such as motor threshold, motor evoked potential (MEP) and the subsequent ‘silent period’ (cortical inhibition phenomenon) for functional outcome at 6 months?
5. Which muscles of the paretic limb (i.e., intrinsic hand muscles, finger and wrist extensors or upper arm muscles) are most sensitive and specific in predicting functional outcome at 6 months post stroke?

Project B2: kinematics

*Research questions:*

1. Can functional improvement of the paretic arm during the first 6 months be explained by behavioural strategies to compensate for deficits (e.g., bending and rotating the trunk) in a standardized reaching task?
2. What is the relation between kinematic changes observed during functional recovery and dynamic changes in cerebral motor networks?

Project B3: haptic robot

*Research questions*

1. How and to what extent do endpoint mechanical behaviour (at the wrist joint) and peripheral reflex chain properties change during follow-up after stroke?
2. Does CIMT or EMG-NMS affect endpoint mechanics and peripheral reflex chain properties?
3. How do endpoint mechanics relate to upper limb performance in executing a reaching task?
4. How are these parameters and changes in parameters in the course of time influenced by CMIT or EMG-NMS?

*2.3 Project C: longitudinal relations. Objective: analysing the longitudinal relations between the results of project B.*

Relatively poor recovery profiles in terms of neurological deficits (FM-score, MI-arm score, somatosensory deficits and neglect) and ARAT may be associated with: 1) the use of compensation strategies (expressed as bending and rotating the trunk towards an object) and 2) the amount of contra-lesional activation in fMRI and TMS (i.e., recruitment of cerebral activity). Those patients showing a normalization of ARAT outcome (i.e. > 53 points) are expected to show: 1) normalization of kinematics and 2) focusing of cortical activity on the original physiological areas involved (i.e. M1, SMA, CC dPMA and vPMA) and intactness of corticospinal tract on TMS.

*Research question*:

How are improvements in dexterity longitudinally related to observed changes in neurological impairments, kinematics and biomechanics and to changes in cortical activation patterns as revealed by fMRI and TMS?

# 3. Clinical outcome variables

Demographics as well as medical history and social status will be recorded of all subjects per project (see appendix). Furthermore, co-morbidity will be assessed from the medical record using the cumulative illness rating scale (CIRS), dexterity will be determined by the Edinburgh Handedness Inventory (EHAI), and a cognitive screening will be performed by the Minimal Mental State Examination (MMSE)

*3.1 Project A: early intervention*

###### Primary outcome measure: effect of the intervention

ARAT:

The Action Research Arm Test (ARAT) will serve as the primary measurement of outcome in both A1 and A2 EXPLICIT trials. The ARAT test is a performance test which assesses the ability to perform gross movements and the ability to grasp, move and release objects differing in size, weight and shape is tested. (Lyle, 1981) The original test consists of 19 items, rated on 4-point ordinal scales (0 to 3). By removing 4 items, a hierarchical 1-dimensional scale has been constructed (Van der Lee et al, 2002). The ARAT has been shown to be valid, reliable and responsive (De Weerdt et al, 1985; Van der Lee et al, 2001). The minimal clinically important difference (MCID) will be set at about 10% of the range of the scale, i.e. 6 points (Van der Lee et al, 1999). For project A2, return of dexterity will be defined as 10 points or more on the ARAT (Kwakkel et al, 2003).

Secondary outcome measures: detecting confounders, comparing groups and tracing changes per patient in time (see appendix)

Ashworth Score (AS)

The Ashworth score is a clinical test by which muscle tone is assessed manually around elbow and wrist.

Wolf Motor Function Test

An extended test of motor function. It is performed only at 3 and 6 months follow up.

Barthel Index

A widely used scale assessing performance of activities of daily living. The Barthel index is scored by the assessor without the need of physical presence of the patient

Frenchay Arm Test (FAT)

A five point scale to test arm function

National Institute of Health Stroke Scale (NIHSS):

A graded neurological examination rating speech and language, cognition, visual field deficits, motor and sensory impairments, and ataxia. (Goldstein et al, 1997)

Motoricity Index-arm and leg (MI):

This test will be used to measure strength in upper and lower extremities (Collin et al, 1990). Higher scores represent greater strength in the upper limb. This instrument reliably and validly assesses the presence of paresis in stroke patients by testing 6 functions rated from 0 to 100 points for each limb (Collin et al, 1990; Kwakkel et al, 1999).

Brunnstrom Fugl Meyer arm/hand test (FM)-arm:

The Fugl Meyer arm score is a reliable and valid motor performance test consisting of 33 tasks performed by the affected upper limb (Badke et al, 1983; Duncan et al, 1983). The FM-arm test evaluates the ability to make movements outside the synergistic pattern. Performance on each task is rated as 0, 1 or 2, with higher ratings representing better performance. The FM-arm measure will be used as the sum of 33 ratings (possible range 0 to 66 points).

O-Letter Cancellation Test (LCT):

Visual inattention will be evaluated by the letter cancellation test (LCT). The O-letter cancellation task has shown high test–retest reliability, ranging from 0.78 to 0.90 (p<0.001) for the number of omissions on the sound and neglected field sides, respectively (Kwakkel et al, 1999).

Nine Hole Peg Test (NHP):

The Nine Hole Peg Test (NHP) is a reliable and valid test that measures manual dexterity (Mathiowetz et al, 1985; Heller et al, 1990). It measures the speed with which a patient grasps and inserts (and removes) 9 pegs into a grid of vertical holes. The test will be discontinued after 150 seconds if the patient is still unable to insert any pegs. The NHPT measure for each hand is calculated by the number of pegs placed per second. The affected as well as the unaffected hand will be measured. Due to floor effects, the NHP is assessed only when the FAT ≥ 5. Reliability and validity have been assessed and norms are available (Mathiowetz et al, 1985; Heller et al, 1987).

Erasmus MC Modification of the (revised) Nottingham Sensory Assessment (EmNSA):

The EmNSA is a 3 point ordinal scale that measures:

1. sharp-blunt discrimination;
2. two-point discrimination and
3. proprioception of upper and lower limb.

The EmNSA will be restricted to the paretic upper limb (i.e., fingers, hand and forearm). With exception of the two-point discrimination, intra- and interrater reliability of tactile sensations, sharp-blunt discrimination and proprioception items are good to excellent (Kappa: 0.58 to 1.00). (Stolk-Hornsveld, et al, 2006)

Stroke Impact Scale version 3.0 (SIS vs. 3.0):

The arm–hand domain of the Stroke Impact Scale (SIS, version 3.0) will be used to evaluate patients’ perceived outcome for the paretic upper limb. Version 3.0 of the SIS is a full-spectrum health status interview that measures changes in 8 impairments, function and quality of life sub domains following stroke and will be used as a secondary outcome measure. (Duncan et al, 2003) Each domain will be analysed separately. The upper limb part of the SIS includes 5 questions about patients’ perceived competency to keep their balance, to transfer, to walk in the house and negotiate stairs, to get in and out of a car and to move about in their own community. Each item is scored from ’not difficult at all’ to ‘cannot do at all’ on a 5-point rating scale. A difference of 5 points (10%) on the ‘hand function’ domain of the SIS is perceived as clinically relevant. (Wolf et al, 2006) The SIS has shown excellent clinimetric properties in terms of concurrent and construct validity, test-retest reliability and responsiveness. (Duncan et al, 1999; van de Port et al, 2007) The SIS will be assessed at baseline and 5, 8, 12 and 26 weeks after stroke. Version 3.0 of SIS has recently been translated into Dutch. (van de Port et al, 2007). The SIS is assessed at 3 and 6 months of follow-up only.

Nottingham Extended Activities of Daily Living (NEADL):

This scale is based on a self-report questionnaire about levels of activity actually performed (Nouri et al 1987) The NEADL consists of 22 items in 4 domains (mobility, kitchen, domestic and leisure). Each item is rated by one of four responses (able, able with difficulty, able with help, unable). The scale has proved to have reasonable hierarchical (ordinal) properties in stroke patients (Nouri et al, 1987). The NEADL will be assessed at baseline and 5, 8, 12 and 26 weeks after randomization.

Motor Activity Log (MAL):

A translated and adapted version of the Motor Activity Log will be used (Van der Lee et al, 2004), which contains the 14 original activities, 11 additional activities, and 1 optional activity chosen by the patient. The list of activities and the rating procedure are shown in Appendix 9. Reliability and validity of the MAL has been proved in a number of studies. (Van der Lee et al, 2004) The MAL will be administered to each applicant and, if available, their caregivers. It will be used to independently rate how well (11-point Quality of Movement [QOM] scale) and how much (11-point amount-of-use [AOU] scale) the paretic arm was used spontaneously to accomplish 30 activities of daily living outside the laboratory (Van der Lee et al, 1999; Uswatte et al, 2005).

*3.2 Project B: cortical reorganisation and compensation strategies*

Primary outcome measures:

fMRI:

focusing of cortical activity on the original physiological areas involved (M1, SMA, CC, dPMA and vPMA) during a standardized wrist flexion-extension movement

TMS:

intactness of corticospinal tract, by early presence of a Motor Evoked Potential (MEP) resulting from transcranial magnetic stimulation in three key arm muscles (*m abductor digiti minimi, m biceps brachii and m. extensor carpi radialis*).

Kinematics:

bending of the trunk during the standard reaching task, as recorded by a 3-D kinematics recording system

Haptic robot:

Velocity induced gain of the peripheral spinal reflex chain and its modulation to changing environmental conditions (damping) as assessed by haptic robotics, system identification and neuromuscular modeling

# 4. Patients and Methods

*4.1 Recruitment*

Subjects will be recruited in equal parts from the 4 participating university medical centres (LUMC, VUmc, RUMC and UMCU).

Patients with a first-ever ischemic lesion in the territory of the MCA, verified by CT and/or MRI scan, will be asked to participate in the first week post-stroke. Patients will receive a Patient Information Letter (Appendix 1) which will explain the background and methods of the study.

After given consent, patients will be randomized. This will include an intake procedure, containing the first assessment of outcome variables. Concealed allocation will be effectuated with sealed opaque envelopes.

The intensity and content of all therapies applied will be recorded in a patient log-book. All groups will receive usual care in terms of lower limb training. Changes in arm and hand function will be measured at fixed times by questionnaires and clinical tests.

*4.2 Coding of the patient data*

After inclusion of the patients into the study each patient will receive a 5 digit code, consisting of a code for the hospital of origin and the first three digits of the patient number. Data processing will take place using the coded patient information.

*4.3 General inclusion criteria*:

- first-ever ischemic lesion in the territory of the MCA, verified by CT and/or MRI scan;
- mono or hemi paresis as determined by a National Institute of Health Stroke Score (NIHSS) ≤ 4
- age between 18 and 80 years;
- able to comprehend and to communicate (MMSE score > 22 )
- written or oral informed consent;
- able to sit for 30 seconds without support;
- demonstrate sufficient motivation to participate in an intensive rehabilitation treatment programme for 3 weeks.

*4.4 Exclusion criteria*

- receiving recombinant tissue plasminogen activator (rTPA, or alteplase) with positive effect
- pacemaker or other metallic implants;
- upper extremity orthopaedic limitations that would affect the results;
- not being able to communicate (i.e. < 4 points on the Utrecht Communication Observation, UCO; Pijfers et al, 1985)
- being disoriented with regard to time and place (i.e., a Mini Mental State Examination (MMSE) score of 22 points or less) (Kwakkel et al, 1999).
- Botulinetoxine injections or medication intake that may influence upper limb function in the previous 3 months
- TMS: a history or family history of epilepsy, craniotomy and/or polyneuropathy
- fMRI: not being able to extend the wrist beyond 10 degrees and the fingers beyond 20 degrees.

*4.5. Allocation to each of the projects:*

#### Project A: early intervention

Project A1: (n=60) Patients with an upper limb deficit with an initially favourable prognosis (NIHSS arm score of 3 or 4 and/or ability to extend the wrist ≥ 10º) will be randomised into 2 groups:

- CIMT and conventional therapy.
- conventional therapy.

Project A2: (n=120) Patients with an initially poor prognosis for recovery of dexterity ((NIHSS arm score of 1 or 2 and/or inability to extend the wrist ≥ 10º) will also be randomized into 2 groups:

- EMG- NMS and conventional therapy;
- conventional therapy.

#### Project B: cortical reorganisation and compensation strategies

30 patients each from the A1 and A2 projects will be asked to participate in project B. They will undergo haptic robot measurements and TMS at set times, while only patients that can extend their wrist beyond 10 degrees and fingers beyond 20 degrees will be asked to participate in the fMRI measurements.

#### Project C: longitudinal relations

There will be no extra measurements for project C. All necessary data are derived from project A and B.

*4.6 Time schedule*

###### Therapy

- CIMT *or* EMG-NMS *or* conventional therapy: week 1 t/m 4

Clinical assessment in Project A:

- ARAT week 1,2,3,4,5,8,12,26
- AS
- MI-arm
- MI-leg
- FM-arm
- LCT
- 9-NHP
- EmNSA
- FAT
- NIHSS
- WMF test week 1,12,26

###### Questionnaires in Project A

- NEADL week 1,2,3,4,5,8,12,26
- MAL
- SIS-3.0 week 1,12 en 26

###### Assessments not requiring direct patient contact

- BI week 1,2,3,4,5,8,12,26

Total time of measurements is approximately 1 hour. When WMF test and SIS are included (at 12 and 26 weeks) total assessment time is approximately 2 hours

###### Additional clinical assessment in Project B

- fMRI week 2, 5 en 26
- TMS week 2, 5 en 26 ( MEP in week 1)
- Haptic robot week 1,2,3,4,5,8,12,26
- Kinematics week 2,3,4,8,26

See also appendix 2

*4.7 Burden and risks*

We are aware of the burden on patients of early intervention and clinical measurements in their early stage of recovery. However, evidence is compelling that in the first weeks after stroke functional outcome is determined. If the treatment has to have any effect on primary outcome it should be applied in the early phase after stroke. Next to the opportunity to evaluate early applied intensive therapy we expect to find answers to fundamental research questions that are vital to our understanding of functional recovery after paresis of the upper limb, which is a prerequisite for future optimal treatment planning.

###### Clinical assessment

Assessment of the patients is performed by a combination of questionnaires and (functional) tasks. There are no invasive measurements. Care is taken that the total time to perform all the required assessments is no more than 1 hour

###### Project A1

The treatment group will receive Constraint Induced Movement Therapy (CIMT) for 5 days a week for three weeks. CIMT consist of immobilizing the unaffected hand with a padded safety mitt (appendix 3) in combination with functional therapy (task oriented exercises, “shaping”) by a Physical Therapist (PT). The exercises last 30 minutes, after which the unaffected arm is immobilized for 3 hours. The risks of the treatment are minimal. The method is not invasive

###### Project A2

EMG-NMS allows for electrostimulation of the affected hand without continuous support of a therapist. Once the patient or his/her spouse has learned to use the apparatus, the therapy can be applied without the need of continuous focusing of the patient. The patient then is free to do other things while the therapy is ongoing. No adverse effects have been reported. EMG-NMS is non- invasive and does not have any side effects.

###### Project B1

fMRI: Patients can experience claustrophobic feelings in an MRI-scanner. They get to wear protective earphones to exclude the beeping and knocking sounds of the apparatus. When patients have no metallic implants such as pacemakers (see exclusion criteria) the risks of MRI are minimal.

TMS: Some patients experience a slight tap on the head during TMS. The stimulation is painless. Although the risk of evoking epileptic insults in patients with a history or family history of epilepsy is precluded by the use of international safety standards, these patients are excluded from the study.

Project B2

Patients will get ample rest between measurements. The method is not invasive.

###### Project B3

Measurements with haptic robots take about 40 minutes including mounting and dismounting. The movement trajectory is a few centimetres. Patients can experience a little muscle-soreness for a few days after the measurement. The method is not invasive.

###### Project C

As there are no extra clinical measurements required, project C poses nu burdens or risks for participating patients

# 5. Statistical analysis

The database will be managed in Excel. The results of the haptic robot will be computed in MatLab first. The analysis will be conducted in SPSS.

Descriptive statistics will be performed on all subjects per project.

Further statistics include correlation and regression of clinical outcome variables (see paragraph 3)

*5.1 Project A: early intervention*

Power analysis:

Project A1: The number of patients is based on a statistical power of 80% (preventing Type II error) with an alpha of 5% (preventing Type I Error) for detecting a meaningful difference of 6 points (i.e., 10%) on the ARAT as the primary outcome measure. (Van der Lee et al, 1999) The population variance (a standard deviation of 8 points in the second week) is based on the findings of a randomized clinical trial in 102 stroke patients with a first-ever MCA stroke (measured with ARAT at 2 weeks post stroke). (Kwakkel et al, 1999; Kwakkel et al, 2003) Controlling for dependency between 5 repeated measurements in the first 5 weeks (test-retest reliability of 0.9 for ARAT based on 5 repeated measurements), a sample-size of 60 patients (including 10% drop outs) should be sufficient.

Project A2: On the basis of a previous cohort study with 101 first-ever MCA strokes (Kwakkel et al, 1999), patients with a poor prognosis have a probability of about 6% to regain some dexterity (ARAT > 9 points) at 6 months post stroke. We assume a proportional difference of 12% in favour of the experimental group. Based on a statistical power of 80% (preventing Type II Error) with an alpha of 5% (preventing Type I Error) and a test–retest reliability of 0.9 for ARAT with 5 repeated measurements, 120 patients should be enough to show statistical significance in favour of EMG-NMS in the poor prognosis group.

*5.2 Project B: cortical reorganisation and compensation strategies*

Power analysis

Project B1: The numbers needed for an fMRI study depend on the signal intensity of the volumes of interest (VOI).Our calculation is based on fMRI measures of Brodmann areas 9 and 46, which are primary association cortices. In these areas, the fMRI activation is assumed to be more difficult than in primary motor and sensory cortices. Therefore, our power analysis uses a conservative estimate of the number of subjects needed. The mean increment of the intensity of the signal in VOI (%) and the standard deviation of the normal signal intensity have both been found to be 1.5, whereas delta 'n' (i.e., the normalized minimal difference between groups that is detectable with fMRI) is estimated to be 1.15. Calculation of the required sample size is based on the formula {N=[2*(z(1-Alpha/2)-z(1-Beta))raise to square*(var(n)/delta)] in which Alpha (preventing type I error) and Beta (preventing type II error) are set at 0.01 and 0.10, respectively. This leads to a sample size of 13 patients in both the control and experimental groups. Based on an expected drop-out rate of 10%, we would need at least 30 stroke victims.

Project B3: Considering the parameter with the largest variability, i.e. reflex gains, a worst-case calculation of sample size, based on earlier measurements on stroke patients using haptic robots, reveals that the number of patients that needs to be measured is 10 in each ‘arm’ of the study, for an alpha of 5% (preventing Type I Error) and a beta of 10% (preventing Type II Error). This calculation is based on a standard deviation of 0.17 Nms/rad. (Meskers et al 2006) The smallest detectable difference will then be 0.12 Nms/rad (two-tailed). Controlling for 10% drop-outs groups of 30 from project A1 en 30 of project A2 should be sufficient.

*5.3 Project C: longitudinal relations*

To investigate the possible longitudinal association between recovery of dexterity on ARAT and covariates, bi-variate longitudinal regression analysis will initially be conducted, including ARAT change scores (i.e. first-order regression) and time-independent covariates at baseline, such as age, gender and lateralization of stroke, as well as change scores of the time-dependent covariates: MI-arm, FM-arm (including wrist), FM-hand, letter-cancellation task and Active Range of Motion (RoM) of trunk rotation and trunk bending during reaching. (Kollen et al, 2005; Kwakkel et al, 2006) Treatment intervention will be used as one of the covariates in the quasi-causal regression model. (Kwakkel, 1999.) Subsequently, standardized regression coefficients will be calculated and a multivariate, first-order regression model to predict functional change on ARAT will be developed. The -2log likelihood ratio test will be calculated to evaluate the necessity of allowing random regression coefficients into the model, and the Wald test will be used to obtain a P value for a particular regression coefficient. A two-tailed significance level of .05 will be used for all tests.

# 6. Time schedule for the project

July 2007-February 2008: Formulating research protocol

Researching background literature

Testing validity of test battery for clinical measurements

Training assessors and researchers in EXPLICIT

February 2008 Protocol to Medisch Ethische Commissie LUMC

February 2008 – June 2008 Submission METC protocol in other UMCs

Training therapists of departments for CIMT and EMG- NMS application

Pilot testing kinematics and motor paradigms fMRI protocol

Informing departments neurology of other UMCs

and rehabilitation wards

July 2008 - August 2011: Recruitment of patients

Clinical measurements and questionnaires

Data analysis

August 2011-February 2012 Clinical measurements and questionnaires

Data analysis

Elaboration of data

Writing scientific articles and thesis

# 7. Financial data

See Appendix 5 for the Budget.

ZON MW will contribute considerably in the research costs of EXPLICIT. The LUMC will pay the salary of one additional OIO.

# 8. Knowledge transfer

The progress achieved by the EXPLICIT programme will be made available by sending newsletters and flyers to the four rehabilitation centres and the university hospital departments of rehabilitation, radiology and neurophysiology

EXPLICIT will produce five PhD dissertations relating to TMS (UMC St. Radboud Nijmegen), fMRI (UMC Utrecht), coordination dynamics (VUmc Amsterdam) and two dissertations on upper limb stiffness (LUMC and TU Delft)

# 9. Literature

Badke et al, Phys Ther 1983;63:13-20

Bolton DA et. al, J Neurol Sci. 2004 Aug 30;223:121-7.

Cirstea MC and Levin MF. Brain. 2000 May;123; 5):940-53.

Cirstea MC et al, Exp Brain Res. 2003 Aug;151(3):289-300.

Collin C et al, J Neurol Neurosurgery & Psychiatry 1990;53:576-580

Conforto AB et al, Ann Neurol. 2002;51(1):122-5.

De Weerdt W et al, Physiotherapy Canada. 1985;37:65–70.

Dobkin BH. N Engl J Med. 2005 Apr 21;352(16):1677-84.

Duncan PW et al, Phys Ther 1983;63:1606-1610

Duncan PW et al, Arch Phys Med Rehabil. 2003;84:950-63.

Duncan PW et al, Stroke 1999;30:2131-2140.

Dijkhuizen RM et al. Proceedings of de National Academic of Sciences of de United States 2001;98:12766-71

Geurts AC et al, Gait Posture 2005;22(3):267-81.

Goldstein LB et al **Stroke. 1997;28:307-310**

Heller A et al, J Neurol Neurosurg Psychiatry. 1987;50:714-9.

Kimberley TJ et al, Exp Brain Res. 2004 Feb;154(4):450-60.

Kwakkel G, Dynamics in functional recovery after stroke. Thesis, 1999.

Kwakkel G et al. Stroke 2003;34:2181-6

Kwakkel G et al, Stroke 2004a;35;2529-2536.

Kwakkel G et al. Rest Neurol Neurosci 2004b; :281-99

Loor HI et al, Neuroepidemiology 1999;18:75-84.

Lyle RC, Int J Rehabil Res. 1981;4:483-492.

Mathiowetz V et al, Arch Phys Med Rehabil. 1985;66:69-74.

Nakayama H et al, Arch Phys Med Rehabil. 1994;75(4):394-8.

Nouri FM, Clin Rehabil 1987;1:301-5

Nudo RJ et al, Muscle Nerve. 2001 Aug;24(8):1000-19.

Pijfers EM etal, Het Utrechts Communicatie Onderzoek. Westervoort: 1985.

Ruwaard D and Kramers PGN (eds.). Volksgezondheid Toekomst Verkenning. De som der delen. Utrecht: RIVM, 1997.

Stephan KM et al, Adv Neurol. 1997;73:311-20

Schaechter et al, Neurorehabil Neural Repair. 2002;16(4):326-38

Stolk-Hornsveld F et al, Clin Rehabil. 2006; 20:160-172.

Sunderland A et al, Neuropsychol Rehabil. 2005;15(2):81-96.

Unswatte et al, Stroke 2005; 36:2493-2496.

van der Lee JH et al, Stroke. 1999;30: 2369–2375.

van der Lee JH et al, Clin Rehabil. 2002;16:646–653.

van der Lee JH et al, Arch Phys Med Rehabil. 2001;82:14–19.

Van der Lee JH et al, Stroke. 2004 Jun;35(6):1410-4.

van de Port IG et al, Am J Phys Med 2007, in press

Wolf SL, et al JAMA. 2006; 1;296(17):2095-104.
